# Kalirin-7 is a Key Player in the Formation of Excitatory Synapses in Hippocampal Neurons

**DOI:** 10.1100/tsw.2010.148

**Published:** 2010-08-17

**Authors:** Xin-Ming Ma

**Affiliations:** Department of Neuroscience, University of Connecticut Health Center, Farmington, CT, USA

**Keywords:** synaptogenesis, dendritic spine, plasticity, estrogen, cocaine, Rho GEF, postsynaptic density

## Abstract

Kalirin-7 (Kal7), a major isoform of Kalirin in the adult rodent hippocampus, is exclusively localized to the postsynaptic side of mature excitatory synapses in hippocampal neurons. Kal7 interacts with multiple PDZ domain—containing proteins through its unique PDZ binding motif. Overexpression of Kal7 increases spine density and spine size, whereas reduction of endogenous Kal7 expression by small hairpin RNA (shRNA) causes a decrease in synapse number and spine density in cultured hippocampal neurons. Hippocampal CA1 pyramidal neurons of Kal7 knockout (Kal7) mice show decreased spine density, spine length, synapse number, and postsynaptic density (PSD) size in their apical dendrites; are deficient in long-term potentiation (LTP); and exhibit decreased frequency of spontaneous excitatory postsynaptic current (sEPSC). Kal7 plays a key role in estrogen-mediated spine/synapse formation in hippocampal neurons. Kal7 is also an essential determinant of dendritic spine formation following chronic cocaine treatment. Kal7 plays a key role in excitatory synapse formation and function.

## UNDERSTANDING THE UNDERLYING MOLECULAR MECHANISMS OF SYNAPTOGENESIS IS OF GREAT INTEREST

The majority of excitatory glutamatergic synapses are localized on dendritic spines, which are small protrusions from the dendritic arbor in the brain[1]. Dendritic spines, which are highly dynamic and have very diverse morphology, are believed to constitute a structural substrate of memory[2]. Changes in synaptic plasticity are often related to the changes in spine density and spine size[3,4]. Increases in both spine number[5,6] and spine size[7,8] contribute to long-term potentiation (LTP) and are associated with learning and memory mechanisms[9,10,11,12]. Larger spines contain more AMPA and NMDA receptors in the postsynaptic density (PSD), and spine head size and the number of presynaptic vesicles are well correlated with the size of the PSD[2]. Alterations in spine density, spine size, and excitatory synapse morphology are found in aging, in an enriched environment, after spatial training, after stress, and in some psychiatric and neurological diseases[13,14,15,16,17,18,19,20,21]. A better understanding of the underlying molecular mechanisms of synapse formation and development will contribute to our understanding of learning and memory, and facilitate the development of strategies for treating psychiatric and neurological diseases.

## KALIRIN, A MULTIFUNCTIONAL PROTEIN, IS REQUIRED FOR SPINE FORMATION IN HIPPOCAMPAL CA1 PYRAMIDAL NEURONS

Understanding spine plasticity requires understanding the factors that control the actin cytoskeleton[22,23]. Rho GTPases, which are activated by Rho guanine nucleotide exchange factors (GEFs) and inactivated by Rho GTPase activating proteins (GAPs), are important intracellular signaling switches in the regulation of cytoskeletal organization[23,24,25,26]. The constitutive activation or inactivation of GEFs is a cause of human disease and a handful of GEFs have been found to be mutated in human cancers[24]. Kalirin, a Rho GEF, activates Rac1, RhoA, and RhoG[27,28,29,30,31,32,33,34]. Alternative splicing of the Kalirin gene and the use of multiple promoters generate at least 10 alternatively spliced isoforms encoding functionally distinct proteins that are expressed in a tissue-specific and developmentally regulated manner[35,36,37,38] (Fig. 1). Kalirin is highly expressed in neurons in the central nervous system (CNS)[36]. The highest levels of Kalirin mRNA, as detected with a probe specific to a conserved spectrin region of all Kalirin isoforms, are found in the cerebral cortex, hippocampus, and Purkinje cells. Relatively high levels of expression are observed in the thalamus, caudate putamen, nucleus accumbens, amygdala, and anterior olfactory nucleus. Low levels of Kalirin mRNA are detected in the hypothalamus. Brain areas with high levels of Kalirin mRNA show strong Kalirin-like immunoreactivity (with an antibody specific to the spectrin region). In the hippocampus, Kalirin staining is observed in the soma and dendrites of the CA1-3 pyramidal cell layer, granule cells of the dentate gyrus, and scattered interneurons throughout the hippocampus[36]. Reduced expression of the most major Kalirin isoforms (Kal7, 9, and 12) causes simplification of the dendritic tree and a marked decrease of spine density, with dispersion of postsynaptic density markers and elimination of presynaptic endings in hippocampal CA1 pyramidal neurons[39]. Among these major Kalirin isoforms, Kal9 and Kal12 are highly expressed during embryonic development[30], and play an important role in axon outgrowth in rat sympathetic neurons[32]. Kal12 binding to dynamin 1 and dynamin 2 through its IgFn domain may play a role in coordinating Rho GTPase–mediated changes in the actin cytoskeleton with dynamin-mediated changes in membrane trafficking[40]. Strong Kal12 immunoreactivity was found in the growth cones of cultured hippocampal neurons (Ma et al., unpublished), suggesting a role for Kal12 in axon outgrowth. The functions of Kal9 and Kal12 in hippocampal neurons remain to be elucidated. The following is a brief summary of our current knowledge of Kal7, the major isoform of Kalirin in the adult rodent brain.

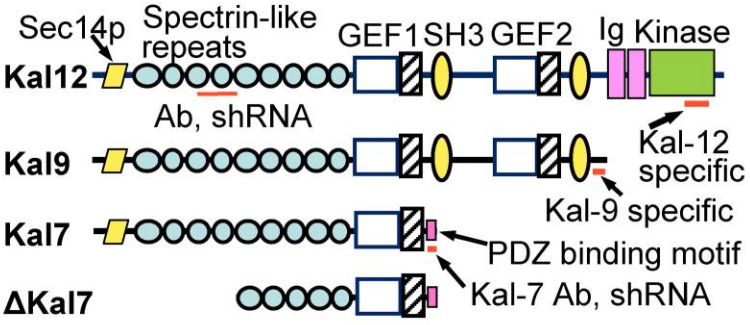

**FIGURE 1.** Alternative splicing generates different isoforms of Kalirin. The largest Kalirin isoform, Kal12, contains one Sec14p domain, nine spectrin-like repeats, two Rho GEF domains, two SH3 domains, two IgFn domains, and one serine/threonine kinase domain. Kal7 contains a unique PDZ binding motif.

## KAL7 IS LOCALIZED TO THE POSTSYNAPTIC SIDE OF EXCITATORY SYNAPSES IN HIPPOCAMPAL NEURONS

Kal7 is primarily expressed in neurons with a high level in the adult hippocampus[39]. Kal7 is distinguished from other Kalirin isoforms by its unique 20-amino-acid C-terminal PDZ binding motif, which allows Kal7 to interact with several PDZ domain–containing proteins[41] (Fig. 1). Kal7 mRNA and protein are found in CA1-3 pyramidal neurons, dentate granule cells, and interneurons scattered throughout the hippocampus[39]. Kal7 activates Rac1, which increases spine density and size[33,42]. Kal7 protein in the rodent hippocampus is undetectable at birth; its levels are extremely low at postnatal day (P) 7 and markedly increase at P14, a key time for synaptogenesis[39,43]. In cultured hippocampal neurons, Kal7-positive clusters are opposed to clusters of stained vesicular glutamate transporter 1 (Vglut1), a marker for excitatory presynaptic terminals (Fig. 2)[44]. Staining for Kal7 overlaps clusters of staining for PSD95 (a PSD marker for excitatory synapses), NMDA receptor subunits NR1 and NR2B, and AMPA receptor subunits GluR1 and GluR2 in both dendritic spines and shafts[44,45]. Synaptic activity regulates Kal7 expression in the postsynaptic side of excitatory synapses in hippocampal neurons because blocking the $GABA_A$ receptor with its antagonist bicuculline increases Kal7 levels in the synapses, accompanied by an increase in the number of excitatory synapses[45]. These data suggest that Kal7 might play an important role in the regulation of excitatory synapse formation.

## KAL7 INTERACTS WITH PROTEINS THAT PLAY A KEY ROLE IN SPINE/SYNAPTIC PLASTICITY

Kal7 interacts with many proteins, such as PSD95, GluR1, NR1, SAP-102, SAP-97, chapsyn-110, neurabin, and spinophillin, in excitatory synapses[41,46]. These proteins play an important role in spine/synaptic plasticity, including assembling trafficking glutamate receptors, signaling complexes, and cytoskeletal remodeling of spines[47,48,49,50]. Kal7 also interacts with neuronal (N)-cadherin, Disrupted-in-Schizophrenia 1 (DISC1), NGF receptor TrkA, and inducible nitric oxide synthase (iNOS)[28,46,51,52]. The interaction of Kal7 and the small GTPase Arf6 suggests that Kal7 may play an important role in Arf6-mediated spine plasticity[53,54]. Kal7 is downstream of cyclin-dependent kinase-5 (Cdk5) and $Ca^{2+}$/calmodulin-dependent protein kinase II (CaMKII) and EphB[33,46,55]. Several key interactors of Kal7 are highlighted below.

- **Cdk5** — Cdk5, a highly versatile kinase, plays a critical role in neurite extension, synaptic plasticity, the formation and function of dendritic spines, learning, and memory[56,57,58,59]. Cdk5 phosphorylates Kal7 at Thr1590, which is localized between the GEF domain and PDZ binding motif, and is the only Cdk5 phosphorylation site in Kal7[33]. Expression of Kal7 with an Ala1590 mutation (T/A, blocking phosphorylation site) causes a decrease in spine size, whereas expression of Kal7 with an Asp1590 mutation (T/D, mimicking phosphorylation) results in an increase in spine size in cultured neurons in comparison to expression of wild-type Kal7[33]. Both Kal7 T/A and T/D increase spine density as well as wild-type Kal7[33]. Alterations of spine size are implicated in learning and memory[12]. The levels of Cdk5 in the PSDs purified from the hippocampus in $Kal7^{KO}$ mice decrease compared to their wild-type controls[60]. These data suggest that Cdk5 regulates Kal7 function during synaptogenesis.
- **CaMKII** — CaMKII, the most abundant protein kinase in the mammalian brain, constitutes the major protein of the PSD in dendritic spines of excitatory neurons[61,62]. It is well established that CaMKII modulates synaptic plasticity, learning, and memory[62,63,64]. Phosphorylation of Kal7 by CaMKII may play an important role in synaptic activity–dependent enlargement of existing spines in cultured neurons[46].

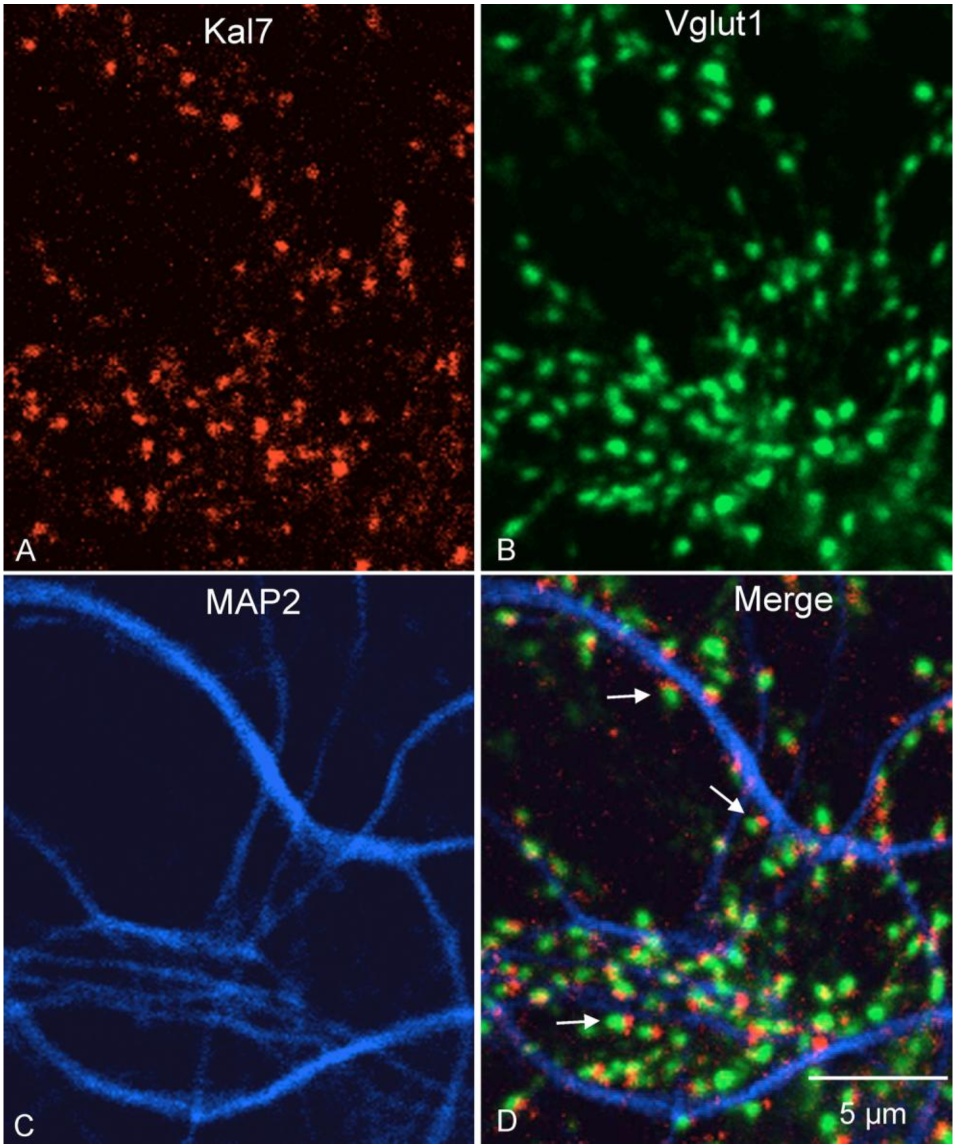

**FIGURE 2.** Kal7 is localized to the postsynaptic side of excitatory synapses in hippocampal neurons. The dendrites of mature hippocampal neurons (DIV30) were fixed with cold methanol and simultaneously stained for Kal7 (A, red), Vglut1 (B, green, a marker for excitatory presynaptic terminals), and MAP2 (C, blue, dendritic marker), showing that Kal7 is localized to the postsynaptic side of excitatory synapses (D, merge). Neuronal preparation and immunostaining were performed as described[44,45].

- **N-Cadherin** — The cadherin-catenin cell-adhesion complex plays a key role in both synapse formation and plasticity[65,66]. Kal7 interacts with N-cadherin[67]. In cultured neurons, N-cadherin and Kal7 colocalize at synapses, and Kal7 function is required for N-cadherin–dependent spine enlargement[67].
- **DISC1** — Recent studies show that DISC1, a strong candidate susceptibility gene for schizophrenia, plays an important role in the regulation of synaptic plasticity[52,68,69]. Schizophrenia patients show a reduction in spine density on their cortical pyramidal neurons[70,71]. DISC1 colocalizes with Kal7 at dendritic spines, and regulates spine structure and function via Kal7. Overexpression of DISC1 causes a decrease in both spine density and spine size, while overexpression of a mutated DISC1, which does not bind to Kal7, does not alter spine density and spine size in cortical neurons in comparison to control neurons expressing GFP only[52] .
- **EphB** — The receptor tyrosine kinase EphB and its membrane-bound ligand ephrinB play an important role in synapse formation and plasticity in the CNS[72,73]. EphB regulates Kal7 function through its phosphorylation of Kal7, and ephrinB-EphB receptor-induced activation of Kal7 causes an increase in the formation of dendritic spines in mature neurons[55].

## KAL7 IS REQUIRED FOR NORMAL SPINE/SYNAPSE FORMATION IN HIPPOCAMPAL PYRAMIDAL NEURONS *IN VITRO* AND *IN VIVO*

Overexpression of Kal7 increases spine density and spine size in hippocampal pyramidal neurons[45] (Fig. 3). Reducing endogenous Kal7 expression by expressing a Kal7 shRNA causes a decrease in synapse number and spine density in cultured hippocampal pyramidal neurons[44,45]. Kal7 shRNA–mediated reduction in spine density was accompanied by a decrease in the frequency of sEPSCs in these neurons (Lemtiri-Chlieh and Ma et al., unpublished). The Kal7 shRNA–induced decrease in spine density is rescued by simultaneous expression of exogenous Kal7 in cultured hippocampal neurons[45]. In addition, a recent interesting study shows that Kal7 is required for microRNA-stimulated spine formation in hippocampal neurons[74]. To evaluate the role of Kal7 *in vivo*, Kal7$^{KO}$ mice were created; these mice grow and reproduce normally[60]. Spine density, spine length, PSD thickness, PSD length, and synapse number decrease in the apical dendrites of Kal7$^{KO}$ hippocampal CA1 pyramidal neurons, which are deficient in LTP and exhibit a decrease in sEPSC frequency. These decreases may be due to decreased levels of NR2B and Cdk5 in the PSDs purified from the hippocampus of Kal7$^{KO}$ mice. Importantly, deficits in spine formation in Kal7$^{KO}$ neurons are rescued by exogenous Kal7[60]. Similarly, a recent study reports that Kal7 plays a key role in spine formation in cortical neurons[75]. These studies show that Kal7 is essential for normal excitatory synapse formation[60,76].

## KAL7 PLAYS A KEY ROLE IN EXCITATORY SYNAPSE FORMATION IN HIPPOCAMPAL INTERNEURONS

Hippocampal interneurons, which are free of dendritic spines, play an essential role in maintaining normal circuits in the CNS, which requires a delicate balance between synaptic excitation and inhibition[77,78]. Low levels of Kal7 are detected at the postsynaptic side of excitatory synapses on the dendritic shaft of hippocampal interneurons[44]. Overexpression of Kal7 increases dendritic branching, and induces the formation of dendritic spines along the dendrites and on the soma of normally aspiny hippocampal interneurons, whereas reducing endogenous Kal7 by shRNA results in a decrease in the number of excitatory synapses on the dendritic shafts of these interneurons[44]. Disruption of interneuron development may contribute to development of psychiatric and neurological diseases[79,80,81]. These studies suggest a role of Kal7 in the development of these diseases.

## KAL7 PLAYS A KEY ROLE IN ESTROGEN-MEDIATED SPINE/SYNAPSE FORMATION IN HIPPOCAMPAL NEURONS

Estrogen increases spine density and excitatory synapse number in both hippocampal CA1 pyramidal neurons of ovariectomized rats *in vivo* and cultured hippocampal neurons *in vitro*[45,82,83]. Spine density[84] and spine size[85] in CA1 pyramidal neurons change across the estrous cycle, peaking during proestrous with its high estrogen levels. However, the underlying mechanisms of estrogen-mediated spine formation are not fully understood. Estrogen replacement increases the intensity of Kal7 staining in both

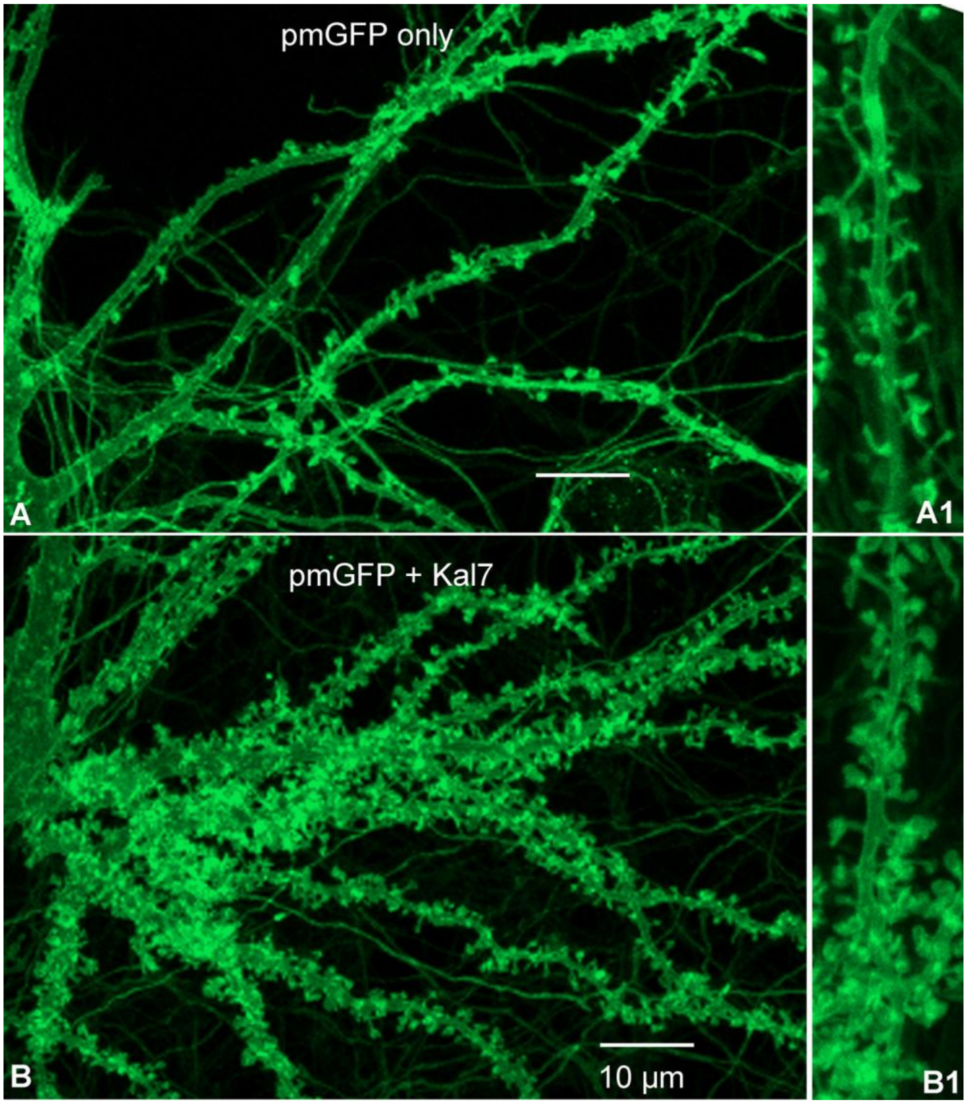

**FIGURE 3.** Kal7 increases spine density in cultured hippocampal neurons when overexpressed. Hippocampal neurons prepared from postnatal day 1 rat pups were transfected with a vector encoding a membrane-tethered version of GFP (pmGFP) alone (A, A1), or vectors encoding both pmGFP and myc-Kal7 in a 1:2 ratio (B, B1) as described[45]. Z-step (0.2 µm) images of live cells were taken at DIV16 with a Zeiss LSM 510 confocal microscope. Expression of Kal7 was verified by Myc staining as described (not shown)[45]. A1 and B1 are high-power images from A and B, respectively. Neuronal preparation, transfection, and immunostaining were performed as described[44,45].

CA1 pyramidal neurons and interneurons in ovariectomized F-344 rats[45]. Estrogen treatment causes an increase in both Kal7 levels at the postsynaptic side of excitatory synapses and in the number of excitatory synapses along the dendrites of pyramidal neurons in cultured hippocampal neurons[45]. The density of excitatory synapses is reduced and estrogen treatment is no longer able to increase synapse formation in hippocampal neurons when endogenous Kal7 expression is reduced using a Kal7-specific shRNA, suggesting a key role of Kal7 in the estrogen-mediated formation of excitatory synapses[45].

## KAL7 PLAYS AN ESSENTIAL ROLE IN SPINE FORMATION FOLLOWING CHRONIC COCAINE TREATMENT

Kal7-mediated spine formation is not specific to hippocampal neurons. Kal7 is expressed in medium spiny neurons of the nucleus accumbens, a brain region involved in drug addiction. Overexpression of Kal7 caused an increase in spine density and spine size, whereas reducing endogenous Kal7 levels caused a decrease in spine density in medium spiny neurons in slice cultures of the nucleus accumbens (Ma et al., unpublished). Chronic cocaine treatment results in a long-lasting increase in dendritic spine density in the nucleus accumbens, but the molecular mechanisms underlying this morphological change are not understood[86]. Repeated cocaine treatment, which increases Kal7 levels in the nucleus accumbens, causes a robust increase in dendritic spine density in medium spiny neurons of the nucleus accumbens core in wild-type mice, but not in Kal7$^{KO}$ mice. In behavioral tests, Kal7$^{KO}$ mice show increased locomotor sensitization in comparison to wild-type controls[87]. Enhanced sensitivity to cocaine in Kal7$^{KO}$ mice suggests that the increase in dendritic spines, normally seen after repeated cocaine, is protective and helps to prevent even more locomotor activity if the spines had not increased. This may also correlate with the decrease in Cdk5 and NR2B seen in Kal7$^{KO}$ mice[60], since lowered Cdk5 and NR2B both enhance locomotor sensitization to stimulant[88,89]. In conclusion, Kal7 plays an essential role in chronic cocaine treatment–induced spine formation, and is necessary for the normal behavioral response to cocaine.

## KAL7 MAY BE RELATED TO PSYCHIATRIC AND NEUROLOGICAL DISEASES

Dendritic spine density and/or spine morphology are altered in psychiatric and neurological diseases, and recent studies suggest a role for Kal7 and its interactors in specific diseases[20,90,91,92,93,94,95]. Kalirin expression increases in the rat hippocampus after electroconvulsive shock, an animal model for electroconvulsive treatment (ECT), an effective therapy for drug-refractory depression[96,97]. Spine density decreases in the hippocampus in animal models of depression[98] and ECT treatment causes an increase in synapse number in hippocampal neurons[99]. These studies suggest that Kal7 may play a role in the ECT-induced increase in synapse number and, thus, in ECT therapy for depression. Several clinical studies suggest that alterations in Kalirin expression may lead to disease. Neuropsychiatric disease is associated with reduced excitatory synaptic connectivity[100,101]. Decreased Kalirin levels are accompanied by a decrease in spine density in the cortex of schizophrenic patients[70,71,102]. Kal7$^{KO}$ mice show decreased anxiety-like behavior and impaired acquisition of a passive avoidance task[60]. Kalirin$^{KO}$ (deletion of major Kalirin isoforms) mice exhibit locomotor hyperactivity and deficits in working memory, sociability, and prepulse inhibition[60,75]. These behaviors are similar to those described for neuropsychiatric disease[103,104,105]. Disturbances in the DISC1–Kal-7 complex–mediated synaptic maintenance may contribute to the onset of neuropsychiatric disease[52]. Kalirin levels are decreased in the hippocampus of Alzheimer’s disease patients[106,107]. The interaction of Kalirin and huntingtin-associated protein 1 (HAP1) suggests that Kalirin is relevant to Huntington’s disease[108]. High levels of Kal7 are found in the CA2-3 hippocampal areas, which are relatively ischemia resistant, suggesting a role of Kal7 in the regulation of ischemic signal transduction[109]. A more recent study indicates that Kalirin is a novel genetic risk factor for ischemic stroke[110].

## CONCLUSIONS AND FUTURE DIRECTIONS

Kal7 is required for synapse formation and function in hippocampal neurons *in vitro* and *in viv*o. Kal7 plays a key role in the mechanisms of spine/synapse formation induced by estrogen and chronic cocaine exposure. Future studies will investigate the underlying molecular mechanisms of Kal7-mediated spine/synapse formation and its contributions to learning and memory, and determine the role of Kal7 in the development of psychiatric and neurological diseases in which normal dendritic spines are altered. Conditional gene deletion approaches will be used to alter Kal7 expression in specific areas or neuronal subpopulations, such as hippocampal CA1 region and nucleus accumbens core.

## ACKNOWLEDGMENTS

This work was supported by grants DK32948, DA22876, and MH086552 from the NIH. Thanks to Drs. Betty Eipper, Dick Mains, Zhao-wen Wang, and William Shoemaker for their critical readings and comments.
